# Novel recombinant coxsackievirus B3 with genetically inserted basic peptide elicits robust antitumor activity against lung cancer

**DOI:** 10.1002/cam4.3143

**Published:** 2020-05-27

**Authors:** Ligang Cai, Zhiyi Liu

**Affiliations:** ^1^ Tongji Medical College of Huazhong University of Science and Technology Wuhan Hubei China; ^2^ Wuhan Boweid Biotechnology Co., Ltd. Wuhan Hubei China

**Keywords:** coxsackievirus B3, cytokines, immune responses, lung cancer, Oncolytic virus therapy

## Abstract

Cancer therapy that utilizes oncolytic virus may offer an exciting alternative, and coxsackievirus B3 (CVB3) is a potent oncolytic virus. This study was to assess the oncolytic activities of novel recombinant CVB3 with genetically inserted basic peptides in lung cancer. Recombinant CVB3 was produced in Vero cells, with or without genetically inserted basic peptides. In vitro and in vivo experiments with nude mouse models bearing human lung carcinoma xenografts were performed to examine the antitumor activities. Cytokines and immune responses to the recombinant CVB3 were determined in cynomolgus monkeys. Recombinant CVB3 with genetically inserted basic peptides was associated with significantly higher pH values within tumors. Mice treated with recombinant CVB3 showed significantly less tumor progression, and recombinant CVB3 with genetically inserted basic peptides appeared to enhance tumor suppression. Recombinant CVB3 was associated with significantly less proliferation of various lung cancer cells without affecting proliferation of normal lung fibroblasts. The cytokine profiles of the cynomolgus monkeys were comparable among control group (normal saline solution) and those given recombinant CVB3 with or without fused basic peptides, with no induction of excessive cytokine or immune responses. In conclusions, recombinant CVB3, especially those with fused basic peptides, possess strong antitumor activities without eliciting excessive immune responses.

## Graphical abstract

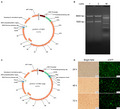

## 1 INTRODUCTION

Oncolytic virus therapy is an exciting alternative treatment for cancer that is based on selective replication and direct induction of death of cancer cells, without causing damage to noncancerous normal tissues.^1^, ^2^, ^3^, ^4^, ^5^, ^6^, ^7^ Oncolytic viruses comprise natural or genetically engineered DNA and RNA viruses.^8^, ^9^ The past several decades has witnessed rapid progress in understanding the mechanisms of oncolytic viruses and their application to cancer treatment.^4^, ^6^, ^10^ Notably, the first genetically modified oncolytic viral therapy, namely IMLYGIC (talimogene laherparepvec), was approved by the United States Food and Drug Administration for the treatment of patients with melanoma, a serious form of skin cancer.^11^ IMLYGIC was developed using a genetically engineered herpes simplex virus type 1 (HSV1). It selectively replicates within melanoma cells and induces cancer cell lysis or death. Aside from IMLYGIC, a number of oncolytic viruses have been under investigation for their therapeutic potential, including coxsackievirus, Newcastle disease virus, measles virus, and others.^10^, ^12^, ^13^, ^14^, ^15^

Coxsackievirus B3 (CVB3) is a member of the enterovirus family and is a known infectious pathogen for viral myocarditis in humans. CVB3 is single‐stranded RNA virus that is approximately 7.4 kb in length. CVB3 replicates in the cytosol of host cells without a DNA phase, and has a highly lytic nature. More importantly, CVB3 selectively replicates within and destroys cancer cells.^3^, ^16^ For its specific characteristics, CVB3 has been proposed as potential oncolytic virus therapy.^3^, ^16^

Recently, Miyamoto and colleagues^10^ reported that CVB3 selectively replicated in and led to apoptosis of human lung adenocarcinoma cells via the mitogen‐activated protein/extracellular signal‐regulated kinase and phosphoinositide 3‐kinase/Akt signaling pathways. This supported the promise that CVB3 may be a potent oncolytic virus in therapy. However, to date, the benefits, efficacy, and safety of CVB3 treatment of specific cancer, such as lung cancer, has not been established.

In this study, we assessed the antitumor effectiveness of novel recombinant CVB3, with or without genetically inserted basic peptides. Nude mouse models bearing human lung carcinoma xenografts were used for assessment of antitumor activities of recombinant CVB3. Cytokines and immune responses to the recombinant CVB3 were examined in cynomolgus monkeys.

## 2 MATERIALS AND METHODS

### 2.1 Cell lines and cultures

Three human non–small cell lung carcinoma cell lines (GLC‐82, A549, and NCI‐H460) were purchased from JOINN Laboratories (Beijing, China). One human small‐cell lung carcinoma cell line NCI‐H1299 was obtained from the Cell Bank of Chinese Academy of Sciences (Shanghai, China). All the lung cancer cells were cultured in Dulbecco's modified Eagle's medium (HyClone) supplemented with 10% (v/v) heat‐inactivated fetal bovine serum (FBS) (Everygreen, Hangzhou, Zhejiang, China) and 100 U/mL penicillin and 100 μg/mL streptomycin, in an incubator at 37°C with 5% CO_2_.

Human lung fibroblasts were obtained from the Hubei University and served as healthy control cells. The lung fibroblasts were grown in RPMI‐1640 medium, supplied with 10% heat‐inactivated FBS (Every Green) and with 100 U/mL penicillin and 100 μg/mL streptomycin in a 37°C incubator with an atmosphere of 5% CO_2_.

Human cardiac cells were purchased from BeNa Culture Collection (Beijing, China). The cells were cultured in RPMI‐1640 (HyClone), supplied with 10% heat‐inactivated FBS (Every Green), and with 100 U/mL penicillin and 100 μg/mL streptomycin in a 37°C incubator with an atmosphere of 5% CO_2._

### 2.2 Selection of basic peptides and amino acid sequences

The following 18 amino acid sequences were designed by methods described previously^17^, ^18^ and used to screen for candidate basic peptides: Arg‐Lys‐Arg‐Lys; Lys‐Arg‐Lys‐Arg; Arg‐Arg‐Lys‐Lys; Lys‐Lys‐Arg‐Arg; Lys‐Arg‐Arg‐Lys (rCVB3‐4pep5); Arg‐Lys‐Lys‐Arg; Arg‐Arg‐His‐Lys‐Lys; Lys‐His‐Arg‐Lys ‐His‐Arg; Lys‐His‐Arg‐Cys‐Lys‐Pro; Arg‐Arg‐His‐Lys‐Met‐Lys; His‐Arg‐Lys‐Cys‐Arg‐Lys; Lys‐Arg‐Trp‐Arg‐Lys‐His‐Arg; His‐Lys‐Gly‐Arg‐Lys‐Cys‐Arg‐Val; Lys‐Arg‐Trp‐His‐Lys‐Met‐Arg‐Lys‐His (rCVB3‐9pep); His‐Phe‐Trp‐Arg‐Gln‐Cys‐Ala‐Met‐Lys; Tyr‐Phe‐Pro‐Arg‐His‐Gln‐Lys‐Trp‐Lys; Trp‐Lys‐Tyr‐Arg‐Gln‐Ile‐Ser‐Thr‐Cys; and Arg‐Lys‐His‐Lys‐Met‐Arg‐Lys‐Cys‐His‐Lys.

Two peptides, Lys‐Arg‐Arg‐Lys (4pep5) and Lys‐Arg‐Trp‐His‐Lys‐Met‐Arg‐Lys‐His (9pep), were selected to be genetically fused to create rCVB3‐4pep5 and rCVB3‐9pep.

### 2.3 Preparation of recombinant CVB3 with or without genetically inserted 4pep5 and 9pep

An attenuated CVB3 strain using pUC57‐CBV3 vector was initially generated, which harbors the full‐length wild‐type CBV3 (Nancy strain, GeneBack ID: JX312064) (GENEWIZ). The recombinant expression vectors were then prepared: pVAX1‐CVB3‐4pep5 and pVAX1‐CVB3‐9pep, with inserted sequences of 4pep5 and 9pep, respectively.

In brief, the wild‐type CVB3 was serially passaged, and amplified in Vero cells. Passages 10, 20, and 30 were subsequently tested for virulence. The smallest plaque morphology was used to construct the expression vectors pVAX1‐CVB3‐4pep5 and pVAX1‐CVB3‐9pep by subcloning the sequences containing basic peptides 4pep5 and 9pep fragments, respectively. The successful insertion of these fragments was validated by a combination of enzymatic digestion and sequencing of pVAX1‐CVB3‐4pep5 and pVAX1‐CVB3‐9pep.

Vero cells were purchased from the Cell Bank of Chinese Academy of Sciences (Shanghai, China), and used to produce recombinant CVB3 with the genetically fused 4pep5 and 9pep. To acquire high titers of recombinant CVB3‐4pep5 and CVB3‐9pep oncolytic viruses, the two vectors pVAX1‐CVB3‐4pep5 and pVAX1‐CVB3‐9pep were transfected into the Vero cells.

### 2.4 MTT assays for cell viability

For in vitro cell viability measurement, 3‐(4,5‐dimethylthiazol‐2‐yl)‐2,5‐diphenyl‐2H‐tetrazolium bromide (MTT) assays were performed in the four human lung carcinoma cell lines (GLC‐82, A549, NCI‐H460, and NCI‐H1299), as well as human lung fibroblasts. Twenty‐four hours before treatment, cells were seeded in a 96‐well plate and grown to reach approximately 80% confluence. Cells were infected with recombinant CVB3 at MOI of 6.67 × 10^−6^ 6.67 × 10^−5^, 6.67 × 10^−4^, 6.67 × 10^−3^, 6.67 × 10^−2^, 6.67 × 10^−1^, 6.67 × 10^0^, 6.67 × 10^1^, 6.67 × 10^2^; or exposed to normal saline (NS) or cisplatin (CIS) as the negative and positive controls, respectively. After 72 hours, MTT assays were conducted in accordance with the manufacturer's protocol (VWR Life Sciences Amresco, Radnor, PA, USA). Briefly, the cell culture medium was replaced with 200 μL of MTT (0.5 mg/mL) in cell culture medium containing 10% fetal bovine serum, and cells continued to incubate at 37°C for 1 hour. Supernatants in each group were removed, and 200 μL of dimethyl sulfoxide was added in each well to solubilize the MTT dye. Absorbance was read at 570 nm on a microplate reader. Each condition was tested with 6 replicates and all assays were performed in triplicate. A half‐maximal inhibitory concentration (IC_50_) was calculated in each group. The inhibition rate (IR) was calculated using the following formula: IR = [Absorbance (NS) – Absorbance (CVB3 oncolytic virus)/Absorbance (NS)] × 100%.

### 2.5 BALB/c nude mice and xenograft human lung carcinoma models for assessment of antitumor activities of recombinant CVB3

This study with BALB/c mice was approved by the Ethics Committee of the Institutional Animal Care and Use Committee, and conducted in accordance with the guidelines for the care and use of laboratory animals.

During the experimental procedures with BALB/c nude mice, we made every effort to minimize pain and other sufferings. None of the mice died, and none were killed due to significant weight loss, inactivity, piloerection, inappetence, or other symptoms.

Male BALB/c nude mice were purchased from Beijing HFK BioScience (Beijing, China) and housed in sterile and filtered cages with water and food ad libitum, and in a quarantined room. Experiments involving experimental animals were performed at the Hubei Center of Food and Drug Safety Evaluation (Wuhan, Hubei, China).

To induce and generate xenograft human lung carcinoma models with BALB/c nude mice, the GLC‐82, A549, and H460 human lung carcinoma cells (5 × 10^7^ cells/mL) were injected subcutaneously into the right flank of the mice.

After solid tumor nodes reached the desired tumor node volume (50‐75 mm^3^), 40 BALB/c nude mice (average tumor node volume 60 mm^3^) were chosen and assigned randomly to the following five groups (n = 8 each group): NS (0.1 mL/10 g); CIS (6 mg/kg); and low‐, medium‐, and high‐dose recombinant CVB3 (6 × 10^4^, 6 × 10^5^, and 6 × 10^6^ plaque‐forming units [PFU]/kg, respectively). In the CIS group, BALB/c nude mice were injected with CIS once per week, which was continued for 5 weeks. The dose and frequency of injections were selected on the basis of our preliminary experiments, in which we evaluated the safety of recombinant CVB3 with low virulence in mice. In the NS and experimental groups (low‐, medium‐, and high‐dose recombinant CVB3), mice were administered the assigned treatment once a day for 5 weeks.

### 2.6 Measurement of pH values within tumors in mice

The pH values within the tumors of the mice were measured using a CL‐9D02 Benchtop pH Meter (NK systems, Osaka, Japan) as described previously.^17^ In brief, these measurements of intratumoral pH values were made on the 41st day, and ~10 mm^2^ of tumor surface was prepared, during which care was taken to avoid damaging the blood vessels. Subsequently, the tumor capsule was perforated using a syringe needle, and the reference capillary was placed on the tumor surface. The pH microelectrode was directly inserted into the tumor at a depth of 0.5 to 1.0 mm. The sizes of these tumors ranged 1.3‐1.9 mm in the NS group, and 0.9‐14.5 mm in the cis, rCVB3, rCVB3‐4pep5, and rCVB‐9pep groups. The pH values within the tumors were recorded with the pH meter.

A subcutaneous A549 cell transplanted tumor model of lung cancer in nude mice was established. Fifteen tumor‐bearing animals having uniform tumor volume were screened. The 15 animals with a tumor volume of 45‐70 mm^3^ (average tumor volume of 56 mm^3^) were assigned to Groups 1‐5 at random. Each group of animals were randomly numbered using Excel software and ranked according to the random number from small to large. There were a total of fiev groups, each group having three animals. The administration mode of all groups are Intravenous injection, NS (0.1 mL/10 g); CIS (6 mg/kg); rCVB3, rCVB3‐4pep5, rCVB3‐9pep groups (6 × 10^6^ PFU/kg). The animals in Group Cis were administered once a week, whereas the other four groups were administered once a day. Throughout the experiment, the average body weight of the animals is increased, and there is no significant difference between groups (*P* < .05). After 1‐week observation, the animals were killed on Day 41.

### 2.7 Animal experiments for the virulence of recombinant CVB3 in mice

Male BALB/c mice aged 3 weeks were purchased from Beijing HFK BioScience (Beijing, China) and used for assessment of the virulence of the recombinant CVB3. Mice were randomly allocated into the following 5 CVB3 groups (n = 10, each): wild‐type Nancy strain (CVB3 Nancy); recombinant without genetically fused basic peptide (rCVB); recombinant oncolytic virus with genetically fused basic peptide 4pep5 or 9pep (rCVB‐4pep5 and rCVB‐9pep, respectively); and NS‐negative control. The experimental mice in each group were injected with 0.3 mL of NS containing wild‐type CVB3 (10^8^ PFU/mL), recombinant CVB3 (10^8^ PFU/mL), or 0.3 mL of only NS as the negative control.

Six days after inoculation, all the experimental mice were killed. The cardiovascular tissues were fixed in 10% formalin for histological examinations. The paraffin sections were analyzed by hematoxylin‐eosin staining, and microscopic images were captured by a digital camera. Histological examinations showed injury to the cardiovascular tissues in mice following inoculation of wild‐type CVB3 Nancy‐strain, whereas no such adverse effect was observed in mice treated with NS or inoculated with rCVB3, rCVB3‐4pep5, and rCVB3‐9pep (Suppl. Figure 1).

**FIGURE 1:**
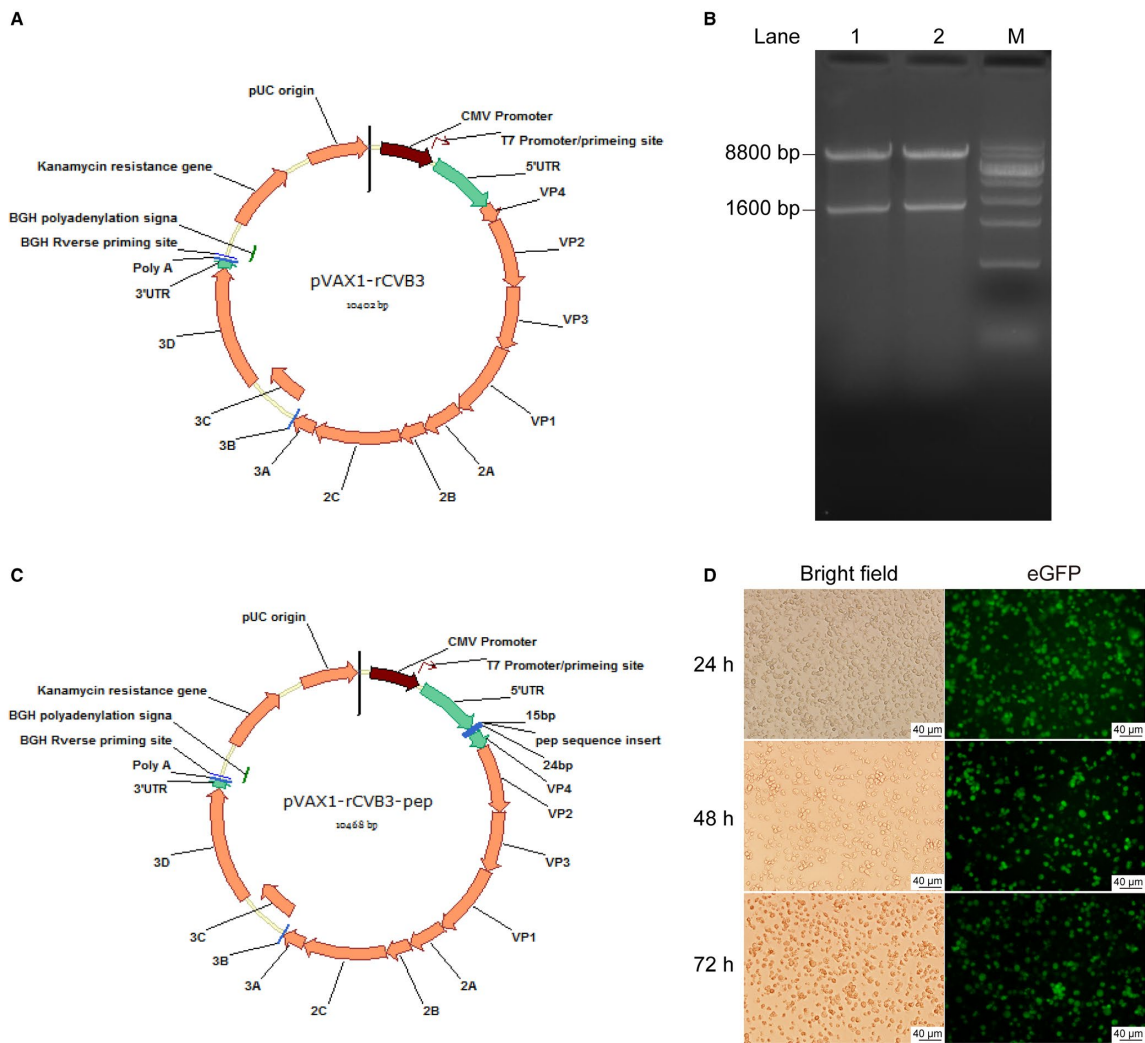
Construction and validation of recombinant CVB3 genetically inserted with basic peptide‐encoding sequences. A, Schematic map of the recombinant CVB3 vector construction; B, Schematic map of the recombinant CVB3 with genetically inserted basic peptide vector construction; C, Electrophoresis analysis of enzymatic digestion products of the two plasmids: pVAX1‐rCVB3‐eGFP‐4pep5 (Lane 1) and pVAX1‐rCVB3‐eGFP‐9pep (Lane 2). NotI and SalI were used. Lane M: DNA standard ladder; D, Expression of eGFP in Vero cells after 48, 72, and 92 h of transfection

### 2.8 Examination of cytokines and immune responses to recombinant CVB3 in cynomolgus monkeys

All study protocols involving the cynomolgus monkeys were reviewed and approved by the Animal Care and Ethics Committee.

As monkeys have similar immune systems to humans and are better than rodents for examining immune responses, they were used for testing effects of recombinant CVB3 on levels of selected cytokines and immune responses. Male and female cynomolgus monkeys (weighing 3.5‐5.5 kg) were purchased from the Hainan Primate Laboratory Animal Development (Haikou, Hainan, China), and housed in an experimental animal facility, which has been fully accredited by the Assessment and Accreditation of Animal Laboratory Care.

The cynomolgus monkeys were allocated randomly to the following four groups (n = 8, each): NS; 0.25 mL/kg) as the negative control; and low‐ (6.25 × 10^6^ PFU/kg/0.25 × 10^8^ PFU/mL), medium‐ (1.25 × 10^7^ PFU/kg/0.5 × 10^8^ PFU/mL), and high‐dose (0.25 × 10^8^ PFU/kg/1.0 × 10^8^ PFU/mL) CVB3. The cynomolgus monkeys were given the assigned treatments intravenously once per day for 13 weeks.

Blood samples were taken from the monkeys at weeks 0, 4, 8, 10, and 13 postinjection and stored frozen for subsequent examinations of selected cytokines. Serum levels of the following cytokines were assayed using an enzyme‐linked immunosorbent assay (ELISA) kit that was specific for the respective cytokines, including interleukin (IL)‐2, IL‐4, TFN‐α, and gamma interferon (IFN‐γ). IgG and IgM responses were determined using the ELISA kit in accordance with the manufacturer's protocol.

The monkeys were observed twice daily during the experimental period for general health, signs of toxicity, mortality, and morbidity. Physical examinations were conducted, and body weights were recorded prior to treatment and weekly thereafter.

### 2.9 Statistical analysis

Statistical analyses were performed with IBM SPSS Statistics 19.0 (SPSS, Chicago, IL, USA). In vitro experiments included triplicate samples for each group and repeated three times. Data are presented as mean ± standard deviation. Differences in mean values were evaluated by Student's *t* test and one‐way analysis of variance with Dunnett's multiple comparison test. *P* < .05 was considered statistically significant.

## 3 RESULTS

### 3.1 Preparation and validation of recombinant CVB3 genetically fused with basic peptides

To construct the expression vectors pVAX1‐rCVB3‐eGFP‐4pep5 and pVAX1‐rCVB3‐eGFP‐9pep, the coding sequences containing 4pep5 or 9pep, respectively, together with enhanced green fluorescent protein (GFP; eGFP), were subcloned to a pVAX1‐rCBV3 plasmid (Figure 1A,B). The insertion of the desired nucleotide sequences was confirmed by enzymatic digestion, sequencing, and expression of GFP. The vectors were digested by specific restriction enzymes and the resulting products were resolved by electrophoresis. The specific bands by base pairs in length were visualized, and were confirmed correct (Figure 1C).

The pVAX1‐rCVB3‐4pep5 or pVAX1‐rCVB3‐9pep vectors were transfected into the Vero cells for packing to produce high titers of recombinant CVB3‐4pep5 and CVB3‐9pep oncolytic viruses, respectively, with the basic 4pep5 or 9pep peptides fused with eGFP. The cells exhibited eGFP at 24, 48, and 72 hours posttransfection (Figure 1D). These observations confirmed that the recombinant CVB3 were genetically fused with the 4pep5 or 9pep basic peptides (Figure 1C,D).

### 3.2 In vitro tumor inhibition effects of recombinant CVB3 in human lung carcinoma cell lines

**FIGURE 2:**
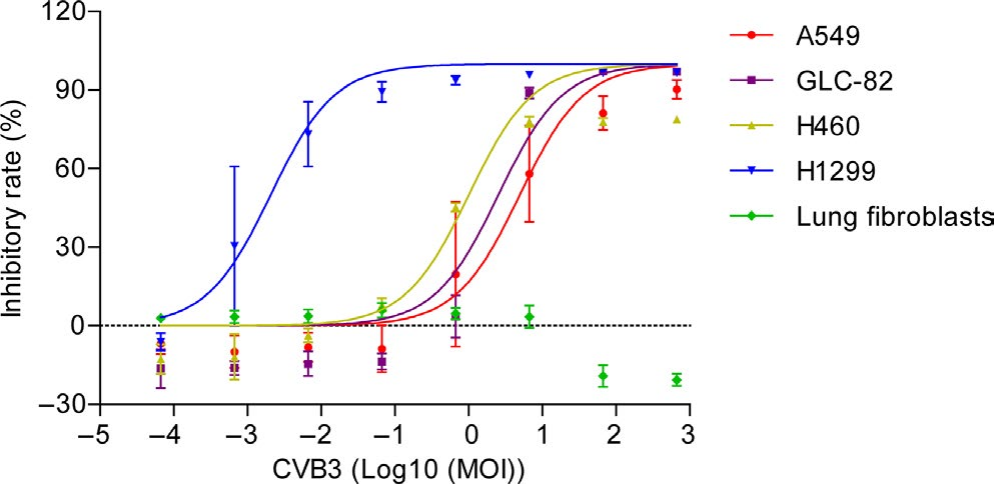
Effects of recombinant CVB3 on cell proliferation of human lung carcinoma cells and normal fibroblasts. MTT assays were performed to determine the effects of recombinant CVB3 on human lung carcinoma cells, including GLC‐82, A549, NCI‐H460, and NCI‐H1299 cells, and normal fibroblasts

We initially examined effects of the recombinant CVB3 on cell proliferation of human lung carcinoma cells, including GLC‐82, A549, NCI‐H460, and NCI‐H1299 cell lines, and lung fibroblasts as a control, using MTT assays (Figure 2). Cells were infected with recombinant CVB3 at MOI of 6.67 × 10^−6^, 6.67 × 10^−5^, 6.67 × 10^−4^, 6.67 × 10^−3^, 6.67 × 10^−2^, 6.67 × 10^−1^, 6.67 × 10^0^, 6.67 × 10^1^, 6.67 × 10^2^; or were exposed to NS as a negative control; or various concentrations of cisplatin (0, 0.00625, 0.025, 0.1, and 0.4 mg/mL) as a positive control.

Compared with the NS group, the recombinant CVB3 exhibited significant antiproliferative effects on human lung carcinoma GLC‐82, A549, NCI‐H460, and NCI‐H1299 cells with > 50% inhibition rates observed at doses of 6.67 × 10^−4^ to 6.67 × 10^2^ (MOI). In addition, the inhibitory effect was dose‐dependent. Of the tested human lung carcinoma cell lines, NCI‐H1299 cells displayed the highest sensitivity to the CVB3‐mediated antiproliferative effect. It was noticed that recombinant CVB3 did not exert any effect on the proliferation of human fibroblasts compared with the NS control group (Figure 2).

### 3.3 Effects of genetically inserted basic peptides on pH values within tumors and antitumor activities of recombinant CVB3 in BALB/c nude mice

**FIGURE 3:**
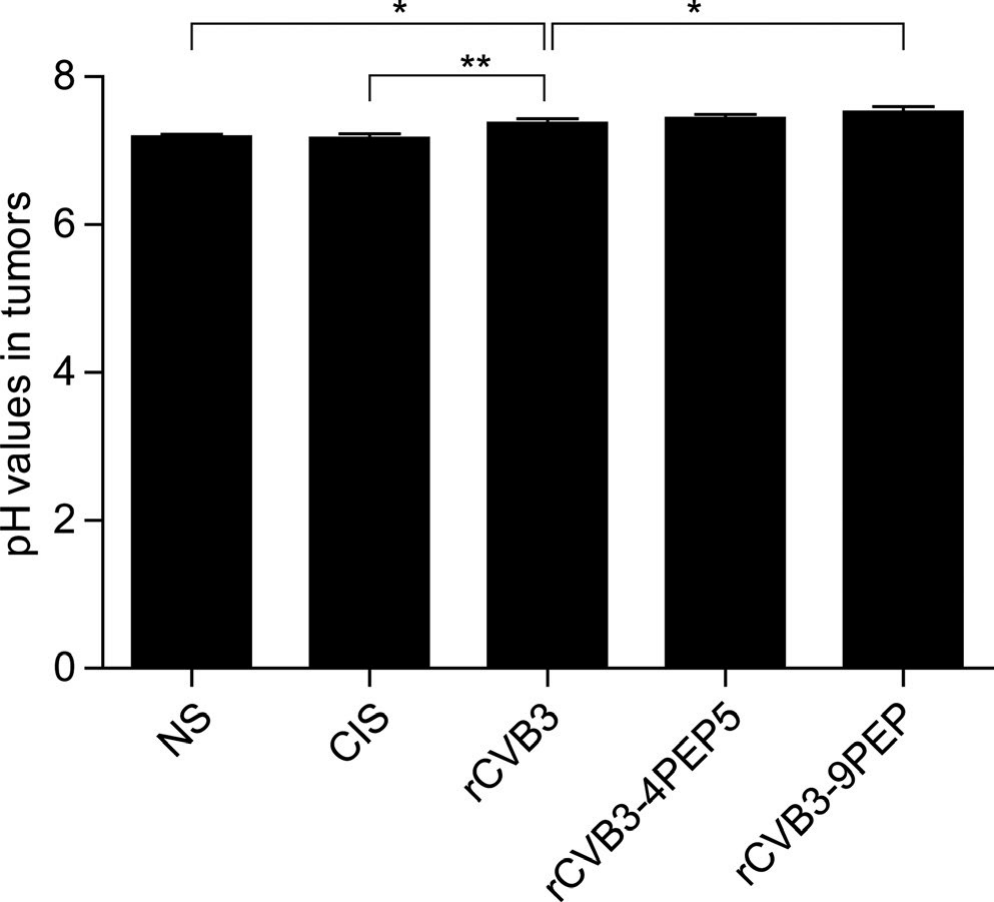
Effects of genetically inserted basic peptides on pH values within tumors in mice. The pH values within tumors in mice were determined and recorded on the pH Meter as described in the Materials and Methods in accordance with the manufacturer's instruction. *Denotes *P* < .05. **Denotes *P* < .01

The effects were determined of recombinant the CVB3 genetically fused basic peptide 4pep5 or 9pep on pH values within tumors (Figure 3). The pH values within tumors in mice treated with the recombinant CVB3 genetically inserted 9pep were significantly higher compared with the tumors of mice with the control recombinant CVB3 (*P* < .05). Although the pH within tumors in the rCVB3‐4pep5 group was slightly higher than that of the control group, the difference did not reach significance.

The tumor suppression effects were examined in vivo in the BALB/c nude mice bearing human lung cancer xenografts, which had been induced by subcutaneous injection of human lung cancer carcinoma GLC‐82. For the model of human lung carcinoma xenografts, the BALB/c nude mice (average tumor nodule 60 mm^3^) were allocated randomly to five groups (n = 8 each group): NS (0.1 mL/10 g), CIS (6 mg/kg), low dose (6 × 10^4^ PFU/kg), medium dose (6 × 10^5^ PFU/kg), or high dose (6 × 10^6^ PFU/kg). Different doses of the recombinant CVB3 were given to the BALB/c nude mice once a day for 5 weeks, whereas NS and CIS were used as the negative and positive controls, respectively.

**FIGURE 4:**
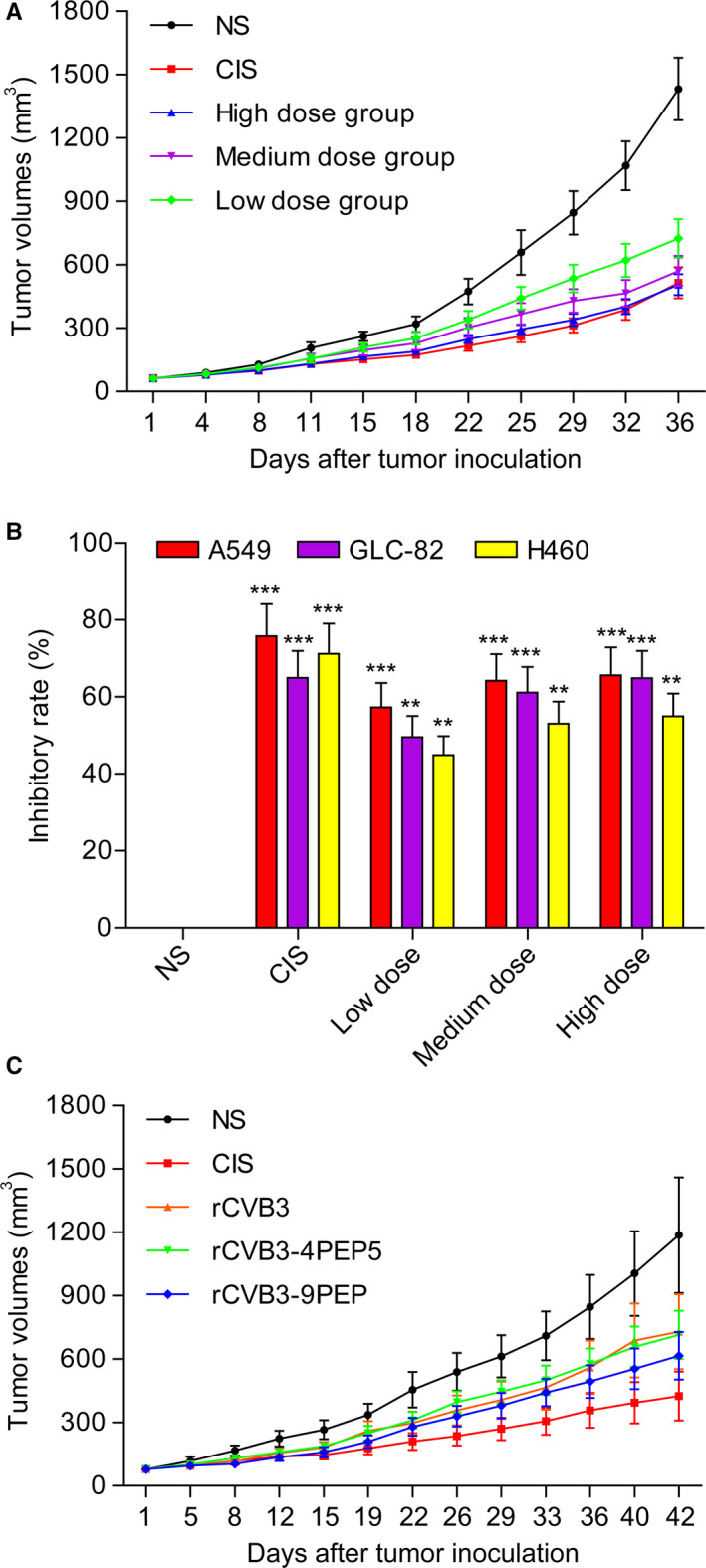
Antitumor activities of recombinant CVB3 with and without genetically fused basic peptides in BALB/c nude mice. A, Antitumor activities of recombinant CVB3 without genetically fused basic peptides in BALB/c nude mice, expressed as tumor volumes. Human lung cancer xenograft mice models were created by subcutaneous injection of human lung cancer carcinoma GLC‐82 cells; B, Antitumor activities of recombinant CVB3 in BALB/c nude mice, expressed as inhibition rate (IR). The BALB/c nude mice bearing GLC‐82, A549, or H460 xenografts were treated with NS, CIS, or recombinant CVB3 at low, medium and high doses; C, Antitumor activities of recombinant CVB3 without and with genetically fused 4pep5 or 9pep basic peptide in BALB/c nude mice. The tumor volumes of the mice bearing human GLC‐82 lung carcinoma xenografts in the NS group rapidly increased as the tumor developed and progressed, whereas the tumor volumes in the mice inoculated with recombinant CVB3 with genetically fused 4pep5 or 9pep basic peptide were lower than that of the controls. **Denotes *P* < .01; ***Denotes *P* < .001

The tumor volumes of the mice bearing human GLC‐82 lung carcinoma xenografts in the NS group rapidly increased as the tumor developed and progressed (Figure 4A). The mice in the low‐, medium‐, and high‐dose groups of recombinant CVB3 (6 × 10^4^, 6 × 10^5^, and 6 × 10^6^ PFU/kg, respectively) exhibited significantly smaller tumor volumes (725.33 ± 260.50, 571.44 ± 203.06, and 506. 67 ± 140.88 mm^3^) compared with those in the NS group (*P* < .05) (Figure 4A). The average tumor volumes of each of the mice were measured. The average tumor volumes of the mice in the NS control group were 1432.36 ± 416.99 mm^3^, whereas those of the mice in the CIS group were 515.34 ± 206.27 mm^3^ (*P* < .05) at the dose of 6 mg/kg body weight.

At the end of the in vivo experiments concerning tumor suppression effects, the mice were killed and lung tumor tissue of each of the mice were excised and weighed. The average tumor weights of the mice treated with NS, CIS, and low, medium, and high doses of recombinant CVB3 were 1.74, 0.7, 1.05, 0.78, and 0.72 g, respectively. The tumor weight inhibition rates of these groups were 60.06, 58.62, 55.24, and 39.94%. The average tumor volumes in the mice treated with CIS, and low, medium, and high doses of recombinant CVB3 were significantly lower than those in the mice treated with NS. The tumor volume inhibition rates of these groups were 64.92%, 49.58%, 61.16%, and 64.88%, respectively.

The BALB/c nude mice bearing GLC‐82, A549 or H460 xenografts were treated with NS, CIS, or recombinant CVB3 at low, medium and high doses. As shown in Figure 4B, the recombinant CVB3 exhibited significant antitumor activities in mice (*P* < .05).

To improve further the tumor suppression effects, we produced recombinant CVB3 fused with basic peptides, which were injected into the nude mice bearing A549 lung carcinoma xenografts. It is of note that the pH values of the tumor tissues were higher in the nude mice treated with recombinant CVB3 fused with basic peptides, relative to the controls (data not shown). Moreover, the treatment with recombinant CVB3 with or without genetically fused basic peptides elicited marked tumor suppression, and the antitumor activity was significantly improved in comparison with recombinant CVB3 without fused basic peptides (Figure 4C). None of the studied mice died of side effects due to the treatments during the in vivo experiments.

### 3.4 Attenuated virulence of recombinant CVB3 with and without genetically fused basic peptides in mice and in human cardiac cells

**FIGURE 5:**
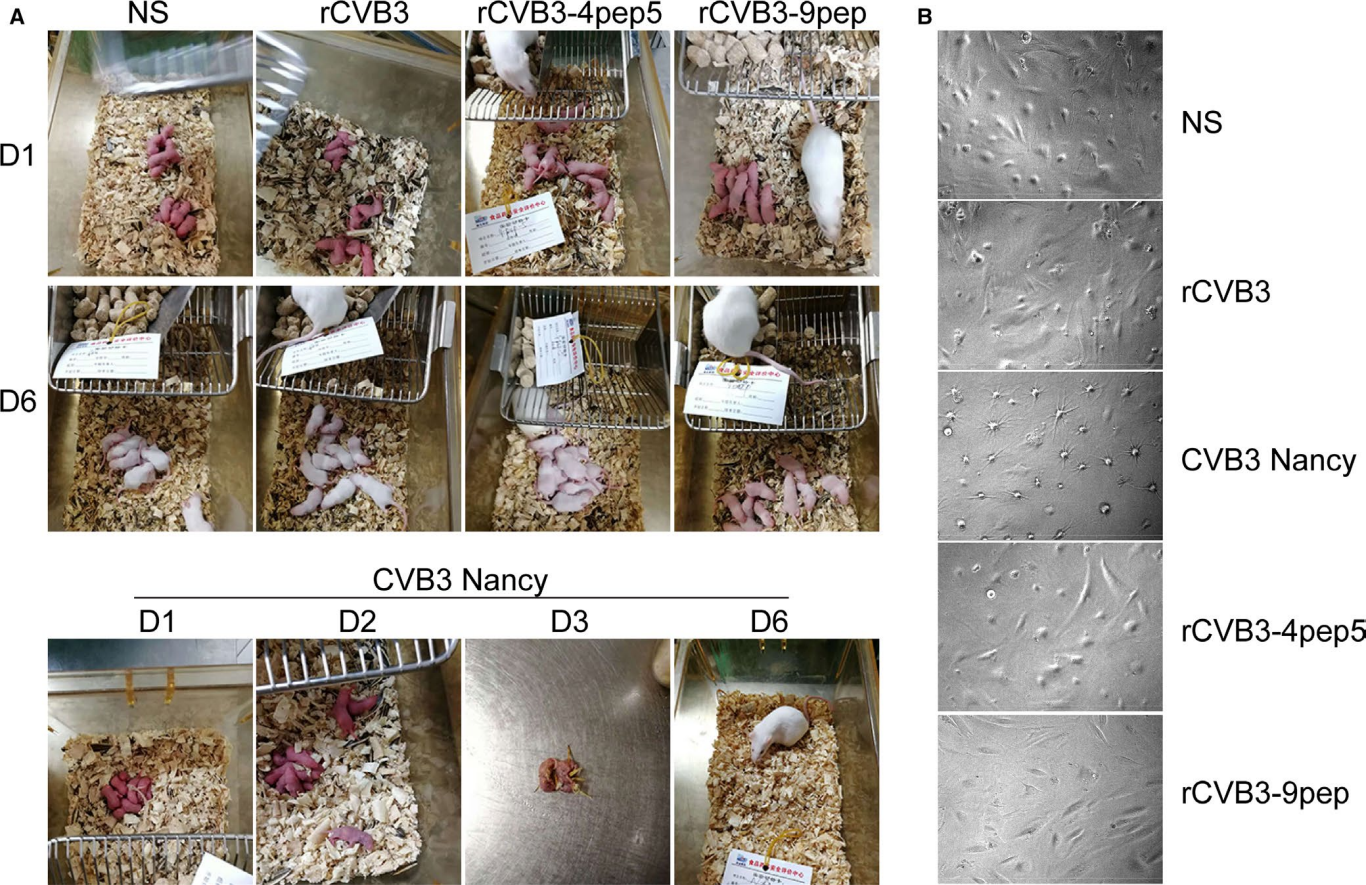
Virulence of recombinant CVB3 with and without genetically fused basic peptides in mice and in human cardiac cells. A, The time of onset of deaths and mortality rates in each group; B, Examination of virulence reduction of recombinant CVB3 under microscope with images captured by a digital camera. Human cardiac cells were also used for Cells were infected with wild‐type Nancy strain CVB3, or recombinant CVB3 (rCVB, rCVB‐4pep5, and rCVB‐9pep) at 10^7^ PFU/mL inoculum, or NS as negative control for 72 h

To determine if the virulence of recombinant CVB3 with and without genetically fused basic peptides in mice was altered, wild‐type Nancy strain CVB3 and recombinant CVB3 (rCVB, rCVB‐4pep5, and rCVB‐9pep) were assessed for their ability to cause lethal infection in the mice (Figure 5A and Suppl. Figure 2). The experimental mice were infected, respectively, with each recombinant CVB3 at the 10^8^ PFU/mL inoculum level or NS as the negative control, and were followed for 6 days. The onset of deaths was at day 3 with a mortality rate of 30%. The mortality rates increased to 60% and 80% at days 4 and 5, respectively, and 100% mortality occurred at day 6 in the mice infected with the wild‐type strain. Notably, in the mice infected with recombinant CVB3, including those with and without genetically fused basic peptides, no death occurred, and the mortality rates were significantly lower than those in the mice infected with wild type.

To evaluate further the reduction in virulence of recombinant CVB3 and the attenuated lethality, human cardiac cells were infected with wild‐type Nancy strain CVB3, or recombinant CVB3 (rCVB, rCVB‐4pep5, and rCVB‐9pep) at 10^7^ PFU/mL inoculum, or NS for 72 hours. The effects were examined under the microscope with images captured by a digital camera (Figure 5B). Apparent attenuation of virulence of recombinant CVB3, with and without genetically fused basic peptides (rCVB, rCVB‐4pep5, and rCVB‐9pep), was observed in the human cardiac cells relative to the wild‐type Nancy strain.

### 3.5 Effects of recombinant CVB3 on levels of selected cytokines and immune responses in cynomolgus monkeys

**FIGURE 6:**
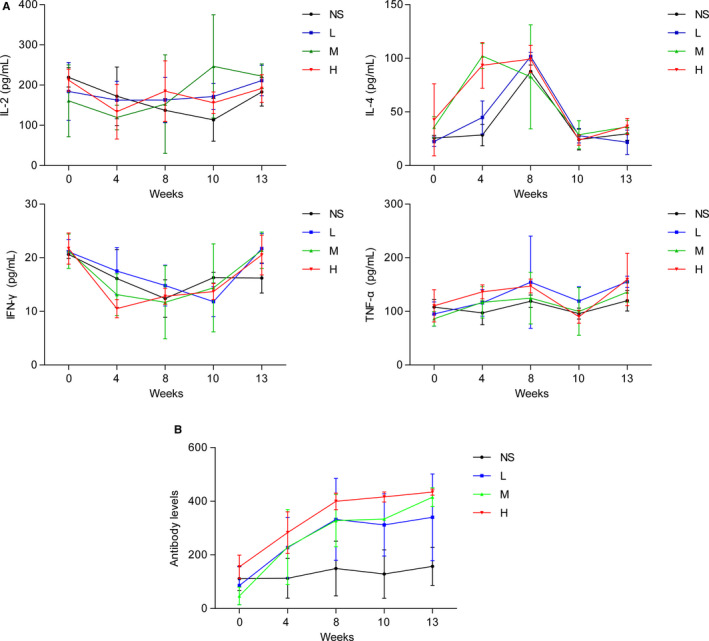
Cytokine and immune responses to recombinant CVB3 in cynomolgus monkeys. L, M, H: low, medium, and high dose, respectively. A, Effects of recombinant CVB3 on levels of serum IL‐2, IL‐4, TFN‐α, and IFN‐γ in the experimental cynomolgus monkeys; B, Effects of recombinant CVB3 on levels of serum IgG and IgM antibodies in the experimental cynomolgus monkeys

The effects of recombinant CVB3 on levels of selected cytokines and immune responses in the experimental cynomolgus monkeys were determined. The tested recombinant CVB3 was administrated to the cynomolgus monkeys at low, medium, and high doses (respectively, 6.25 × 10^6^ PFU/kg/0.25 × 10^8^ PFU/mL, 1.25 × 10^7^ PFU/kg/0.5 × 10^8^ PFU/mL, and 0.25 × 10^8^ PFU/kg/1.0 × 10^8^ PFU/mL) for 13 weeks, whereas NS (0.25 mL/kg) served as a negative control. The serum levels of the selected cytokines (IL‐2, IL‐4, TFN‐α, and IFN‐γ) of the three concentrations of recombinant CVB3 were similar to that of the NS control (Figure 6A). Specific antibodies (IgG and IgM) were initially detected after 4 weeks of treatment with different doses of recombinant CVB3, and increased over the following weeks (Figure 6B), indicating no excessive cytokines or immune responses in the cynomolgus monkeys. In addition to the cytokines, the body weight, clinical signs of toxicity, morality, and morbidity were monitored during the study, and no significant clinical changes were observed in the cynomolgus monkeys.

## 4 DISCUSSION

Lung cancer is the leading cause of cancer deaths in both men and women, accounting for approximately 1.6 million deaths per year worldwide.^17^, ^18^, ^19^, ^20^ This study of the effectiveness of novel recombinant CVB3 has the following major novel findings. First, recombinant CVB3 significantly suppressed the proliferation of GLC‐82, A549, NCI‐H460, and NCI‐H1299 lung cells without toxicity to normal lung cells. Second, the recombinant CVB3 significantly reduced tumor volume of the BALB/c nude mice bearing human lung carcinoma xenografts, suggesting the suppressive actions in the development and progression of lung cancer. Thirdly, recombinant CVB3 with genetically inserted basic peptides was associated with significantly higher pH values within the lung cancer tissues and greater in vivo and in vitro antitumor activities. Finally, the recombinant CVB3, with and without fused basic peptides, did not elicit excessive cytokine and immune responses in the cynomolgus monkeys.

Mouse models bearing human lung carcinoma xenografts and treated with injection of recombinant CVB3 showed significantly less tumor progression, and recombinant CVB3 with genetically inserted basic peptides appeared to enhance tumor suppression.

Tumor microenvironment, including the pH homeostasis, has been shown to be involved in the malignant transformation and growth. In particular, the pH value of solid tumors has been reported to be more acidic than that of normal tissues, and indeed cancer cells may survive and thrives in a highly acidic condition. Acidic condition may contribute to rapid and aggressive progression of solid tumors, for which an alteration in the tumor microenvironment via an elevation of the pH value is proposed to have inhibitory effects on the tumor growth. In this study, the recombinant CVB3 was infused with basic peptides, and we initially hypothesized that the insertion of these basic peptides may increase the pH value and therefore may improve the tumor inhibitory effects. As expected, the pH values of the tumor tissues were higher in the nude mice treated with recombinant CVB3 fused with basic peptides, and their antitumor activity was also significantly improved in contrast to the recombinant CVB3 without fused basic peptides. Furthermore, we showed that genetically inserted basic peptides enhanced tumor suppression, but without introduction of unfavorable excessive cytokine and immune responses. It is worthy noting that, in this study, administrations of rCVB3 without or with genetically inserted basic peptides showed antitumor effects against lung carcinoma xenografts. Although the underlying mechanisms remain elusive, we hypothesize that infections with rCVB3, rCVB‐4pep5, or rCVB‐9pep may restore inherent antitumor immunity. We also postulated that rCVB3, rCVB‐4pep5, or rCVB‐9pep could induce interferons/cytokines, through which mature dendritic cells and natural killer cells could be activated in the microenvironment of the lung tumor tissues.^21^, ^22^, ^23^ Likewise, there is a possibility that the immunostimulatory properties of the rCVB3 on innate immunity may stimulate adaptive immunity responses, which could in turn synergize to promote the oncolytic activities.

To compare the reduction in virulence of rCVB, rCVB‐4pep5, and rCVB‐9pep relative to the wild‐type Nancy strain CVB3, experiments in mice and histologic assays of human cardiac cells during the treatment were performed. Notably, wild‐type Nancy strain CVB3 administration induced lethal infection in mice, whereas rCVB, rCVB‐4pep5, and rCVB‐9pep administration led to marked attenuation of virulence. It may also merit attention that rCVB, rCVB‐4pep5, and rCVB‐9pep administration did not cause severe myocarditis, evidenced via histological examinations of human cardiac cells. This suggests that rCVB, rCVB‐4pep5, and rCVB‐9pep may induce mild side effects, and have potential as acceptably safe oncolytic agents in the treatment of lung cancer.

In addition, the majority of previous studies were conducted in human xenograft tumor models in nude mice or immune‐deficient mice, which lack adaptive immune responses.^10^, ^16^ With the use of cynomolgus monkeys, we analyzed serum cytokines and immune responses to the recombinant CVB3. In the monkeys, the recombinant CVB3 with genetically inserted basic peptide‐encoding nucleotide sequences did not show evidence of changes in the secretion of cytokines (IL‐2, IL‐4, TFN‐α, and IFN‐γ), indicating no excessive cytokine and immune responses.

The study may have potential limitations. As a new class of cancer therapies, oncolytic virus therapy with various types of viruses may have distinct mechanisms of action.^24^, ^25^, ^26^, ^27^, ^28^, ^29^, ^30^ The molecular mechanisms, by which recombinant CVB3 exert antitumor properties against lung cancer, will need to be clarified. Further investigations are underway to gain insight into the underlying molecular pathways. Built upon the exciting results, it may be worth extending the results from mice to monkeys in the future.

Taken together, the findings in this study indicate that recombinant CVB3, especially with fused basic peptides, is a potent oncolytic agents, and thus hold promise as oncolytic virus therapy for lung cancer. The elimination of excessive cytokine and immune responses would significantly improve the safety and minimize the adverse effects associated with oncolytic virus therapy in the treatment of lung cancer.

## CONFLICT OF INTEREST

Ligang Cai declares that there is no conflict of interest. Zhiyi Liu was employed by Wuhan Boweid Biotechnology Co., Ltd., Wuhan, China.

## AUTHORS' CONTRIBUTIONS

LC designed the experiments and wrote the paper; LC and ZL performed the experiments and data analysis. All authors read and approved the final manuscript.

## Supporting information

Fig S1

Fig S2

## Data Availability

The datasets generated and analyzed during this study are available from the corresponding author on reasonable request.
