# Supplementary figures and images for: Novel recombinant coxsackievirus B3 with genetically inserted basic peptide elicits robust antitumor activity against lung cancer
Source: Cancer Med. 2020 May 27;9(14):5210–20. doi: 10.1002/cam4.3143 (PMC7367620; doi:10.1002/cam4.3143)

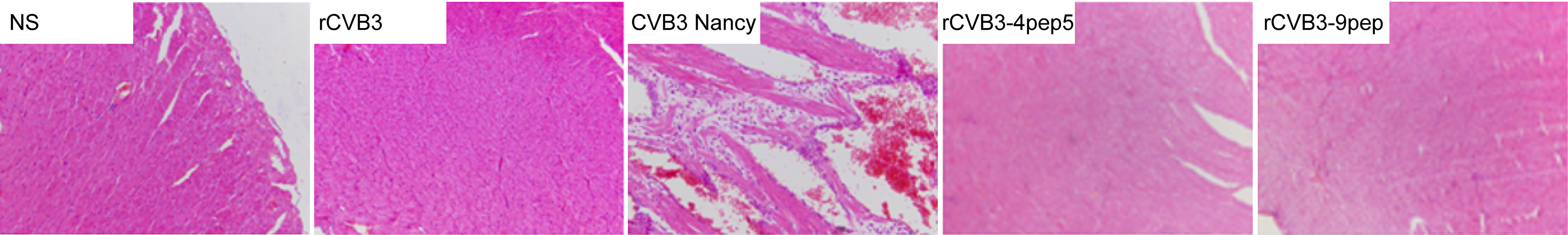

Supplement: Supplementary file 1 — Fig S1 [file CAM4-9-5210-s001.tif]

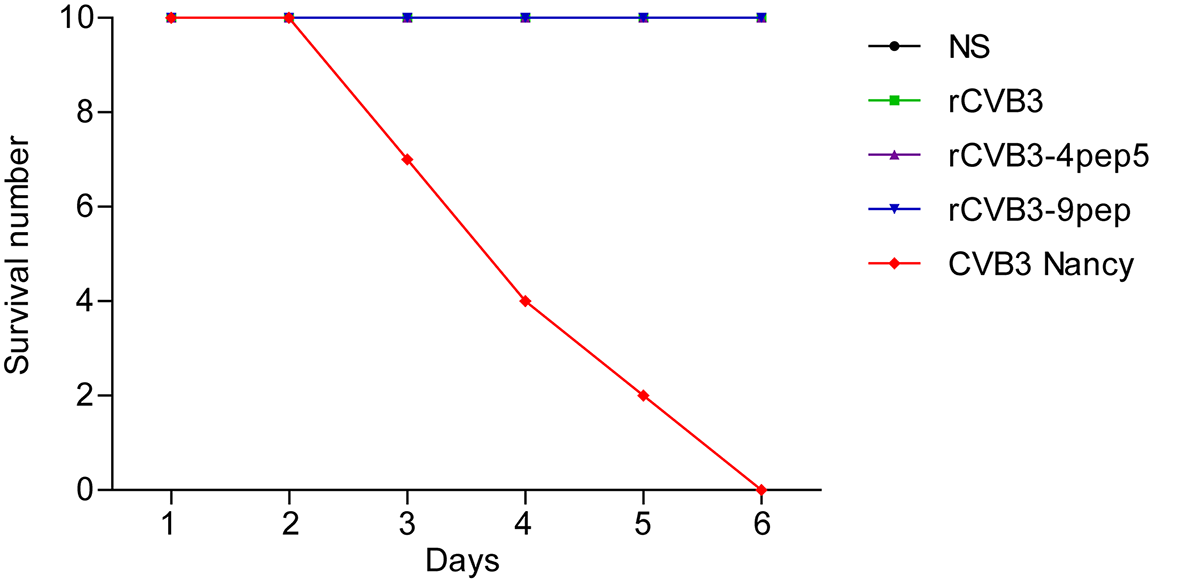

Supplement: Supplementary file 2 — Fig S2 [file CAM4-9-5210-s002.tif]
